# Lens Subluxation after Plasmin and SF_6_ Injections in Rabbit Eyes

**DOI:** 10.1371/journal.pone.0112957

**Published:** 2014-11-14

**Authors:** Wei-Chi Wu, Chi-Hsien Liu, Nan-Kai Wang, Kwan-Jen Chen, Tun-Lu Chen, Yih-Shiou Hwang, Pei-Ju Liao, Lien-Min Li, Chi-Chun Lai

**Affiliations:** 1 Department of Ophthalmology, Chang Gung Memorial Hospital, Taoyuan, Taiwan; 2 Chang Gung University, College of Medicine, Taoyuan, Taiwan; 3 Graduate Institute of Biochemical and Biomedical Engineering, Chang Gung University, Taoyuan, Taiwan; 4 Department of Health Care Administration, Oriental Institute of Technology, New Taipei City, Taiwan and Department of Business Administration, National Taiwan University, Taipei, Taiwan; Children’s Hospital Boston, United States of America

## Abstract

**Purpose:**

To investigate the rate of lens subluxation following plasmin and/or SF_6_ injections in eyes, and whether a subsequent elevated level of vascular endothelial growth factor (VEGF) and vitreous tap would aggravate subluxation.

**Methods:**

Four groups of rabbits were used. Group 1 received an intravitreal injection (IVI) of plasmin and SF_6_ in the right eye; group 2 received an IVI of plasmin in the right eye; group 3 received an IVI of SF_6_ in the right eye; and group 4 received an IVI of balanced salt solution in the right eye. After treatment, IVIs of VEGF were given and vitreous tap was performed three times, followed by clinical observation of lens subluxation and scanning electronic microscope evaluation of the zonular fibers.

**Results:**

After IVIs of plasmin and SF_6_, and VEGF and vitreous tap had been performed one to three times, lens subluxation was noted in 0%, 43%, 71%, 71%, and 86% of the eyes in group 1. After IVIs of plasmin, VEGF, and vitreous tap had been performed one to three times, lens subluxation was noted in 11%, 22%, 44%, 44%, and 67% of the eyes in group 2. The eyes in group 3 and 4 did not show signs of lens subluxation after VEGF IVIs and vitreous tap. Histology confirmed zonular fiber damage in the eyes treated with plasmin.

**Conclusions:**

The incidence of lens subluxation increased following plasmin injections in the eyes, and this was aggravated by the subsequent high VEGF level in the eyes and vitreous tapping. Zonular fibers were disrupted following plasmin treatment. These effects should be kept in mind when using plasmin enzymes in patients with vitreoretinal abnormalities.

## Introduction

Vitreous traction on the retina can be a significant pathological factor in certain retinopathies, including central retinal vein occlusion, pediatric vitreoretinopathy, diabetic retinopathy, age-related macular degeneration (ARMD), and cystoids macular edema [1]–[7]. Several studies have shown that patients with posterior vitreous detachment (PVD), which is characterized by a lack of vitreous traction on the retina, have a better visual prognosis in certain retinopathies such as retinal vessel occlusion and ARMD [1], [4]. Relief of vitreous traction by the induction of PVD would therefore be theoretically helpful for these retinopathies.

Plasmin is a serine protease that mediates the fibrinolytic process and modulates the extracellular matrix [8]. It hydrolyzes a variety of glycoproteins including laminin and fibronectin, both of which are present at the vitreoretinal interface and are thought to play a key role in vitreoretinal attachment [9], [10]. Plasmin enzyme has been proven to cause vitreous liquefaction and PVD [11]–[17]. Pharmacologic vitreolysis with microplasmin (ThromboGenics Ltd., Dublin, Ireland), a truncated form of plasmin, has been reported to increase vitreous diffusion coefficients [18] and oxygen levels in the vitreous [19]. Therefore, plasmin may be useful in treating a variety of retinopathies because it reduces vitreous traction and retinal ischemia.

Several animal studies have shown a good safety profile for plasmin when used in the eye [20]–[23]. However, laminin and fibronectin are structural components of ciliary zonular fibers [24], [25], and the ciliary body also expresses laminin and fibronectin [10]. Theoretically, the intravitreal use of plasmin enzyme may also digest these components and affect the integrity of the zonular fibers. Recently, a commercially available truncated plasmin, ocriplasmin (Jetrea, Thrombogenics Ltd.) has been approved for use in vitreomacular traction and macular holes [26]. There are currently no clinical studies on lens subluxation in patients following the use of ocriplasmin, however, 1 case of drug-related lens subluxation and 1 case of lens instability where drug causality could not be ruled out were reported in the biologics license application for Ocriplasmin submitted to the Food and Drug Administration (FDA) by Thrombogenics. (http://www.fda.gov/downloads/advisorycommittees/committeesmeetingmaterials/drugs/dermatologicandophthalmicdrugsadvisorycommittee/ucm313091.pdf. Accessed 8 September 2014). Since ocriplasmin and plasmin act via the same active site, this effect may be of particular concern when using plasmin or plasmin-associated enzymes in patients with vitreomacular abnormalities. In addition, vascular endothelial growth factor (VEGF) is known to be highly elevated in eyes with a variety of retinopathies including ARMD [27], diabetic retinopathy [28], [29], retinopathy of prematurity [30], [31], and retinal vessel occlusion [29], [32], [33]. Whether further increases of VEGF in eyes after plasmin injection and subsequent vitreous tapping would further affect zonular integrity remains to be investigated.

Taking these factors into consideration, the aim of this study was to investigate the effect of plasmin and/or SF_6_ injections on the integrity of zonular fibers, ciliary processes, and lens subluxation, and whether further increases in VEGF levels and vitreous tapping in the eyes would aggravate these factors.

## Materials and Methods

### Animals

Thirty-four Japanese white rabbits (2.5 kg) were used in this study. Three rabbits died during the procedure of anesthesia, and the remaining 31 rabbits were used for data analysis. The animals were purchased from the Animal Health Research Institute, Council of Agriculture (Executive Yuan, Jhunan, Taiwan), and were housed in the animal care facilities of the Chang Gung Memorial Hospital, Taoyuan, Taiwan. Animal handling was performed in accordance with the regulations of Chang Gung Memorial Hospital for the use of experimental animals and the Association for Research in Vision and Ophthalmology statement for the use of animals in Ophthalmic and Vision Research. This project followed the guidelines and standards of Good Laboratory Practice.

### Grouping of the Animals

The animals were divided into 4 groups with different eye treatments. The right eye of each rabbit in group 1 received a pars plana injection of 1 unit of human plasmin (CalBiochem, La Jolla, CA; 0.1 ml reconstituted in sterile balanced salt solution [BSS]) plus 0.5 ml of 100% SF_6_ in the mid-vitreous cavity (plasmin+SF_6_ group; n = 7). The right eye of each rabbit in group 2 received an intravitreal injection of 1 unit of human plasmin (0.1 ml) only (plasmin group; n = 9). The right eye of each rabbit in group 3 received an intravitreal injection of 0.5 ml of 100% SF_6_ only (SF_6_ group; n = 7). The rabbits in group 4 received a BSS (0.1 ml) injection in the right eye (BSS group; n = 8). The left eye of each animal in these 4 groups did not receive an injection. Anesthesia was performed as previously described [34].

The intravitreal injections were performed 2 mm posterior to the limbus at a 3 o’clock position in the nasal quadrant, and the procedure was performed under a surgical microscope (M691; Wild Heerbrugg, Heerbrugg, Switzerland) with a prism lens. Care was taken to avoid damage to the lens and the retina during injection.

### Intravitreal Injections of Vascular Endothelial Growth Factor (VEGF)

Intravitreal injections of human VEGF (recombinant human VEGF_165_; R & D Systems, Minneapolis, MN) were performed in the plasmin-treated eyes and control eyes to determine whether a high level of VEGF in the eye would affect zonular integrity, thereby inducing lens subluxation. Two months after plasmin and/or SF_6_ or BSS treatment, VEGF (50 ul, 10 ng/ul) was injected into the right eyes of each rabbit to stimulate a high level of VEGF.

### Vitreous Tap

**Figure 1:**
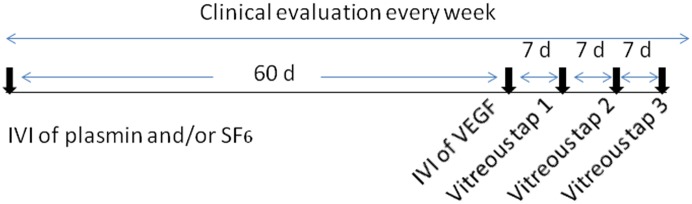
Study design. The time points of treatments and the clinical evaluation. D, days. IVI, intravitreal injection.

Vitreous tap was performed every 7 days after the intravitreal injection of VEGF. In total, 3 taps were performed in each animal. Vitreous tap was performed with a 1 ml syringe attached to a 30 gauge needle. The injection site was 2 mm posterior to the limbus. After the needle was placed in the vitreous, a vitreous tap of 200 µl was performed. Topical antibiotic ointment was used after each tapping. The time points of the experimental procedures are shown in **Figure 1**.

### Clinical Observations

The animals were checked every week to examine whether there were signs of lens subluxation until they were sacrificed. Slit-lamp (SL-15; Kowa, Tokyo, Japan) examinations and indirect ophthalmoscopy (Omega 500; Heine, Herrsching, Germany) were performed to check the status of the cornea, conjunctiva, lens, vitreous, and retina after treatment. Special attention was paid to the lens position in these animals. Lens subluxation was defined as a displaced lens remaining behind the pupil with any space visible between the iris and lens due to incomplete rupture of the zonule after the rabbit’s pupils were fully dilated. In additional to clinical examinations, external photos of all of the eyes before and after the injections were captured by a digital camera and the images were examined using Photoshop (CS5, Adobe Systems Incorporated, San Jose, CA) to verify the severity of lens subluxation. The degree of lens dislocation was classified into 3 broad groups according to a previous publication [35]: (1) mild lens subluxation in which the lens edge uncovered 1% to 25% of the dilated pupil; (2) moderate lens subluxation in which the lens edge uncovered 26% to 50% of the dilated pupil; and (3) and severe lens subluxation in which the lens edge uncovered more than 50% of the pupil.

### Histologic and Electron Microscopic Examinations

Nearly 90 days after the initial intravitreal injections, all of the animals were sacrificed by an overdose of anesthetics. After enucleation, the eyes were opened with a razor blade, which was used to ensure rapid penetration of the fixative. Care was taken to avoid damage to the adjacent lens. The eye button was cut from the sclera about 5 mm away from the limbus. Morphologic examinations of the ciliary processes and the zonular fibers were then performed.

For scanning electron microscopy, the samples were fixed in a mixture of 3% glutaraldehyde and 2% paraformaldehyde in 0.1 M cacodylate buffer, dehydrated through a graded ethanol series, dried in carbon dioxide liquid to the critical point, sputter-coated in platinum, then photographed using an electron microscope (S-5000; Hitachi, Tokyo, Japan). The integrity of the zonular fibers anchored between the lens and the ciliary processes was evaluated by the electron photomicrographs. Two observers who were blinded to group classification interpreted the morphology data.

### Statistical Analysis

Generalized estimating equations (GEE) were used to examine the differences in lens subluxation between groups. The assumptions of applying GEE to the experimental data were checked and appropriate adjustments were performed before GEE modeling. Statistical Analysis System (SAS) software version 9.2 (SAS Institute Inc., Cary, NC) was used for all data analyses. A p value less than 0.05 was considered to indicate statistical significance in this study.

## Results

**Figure 2:**
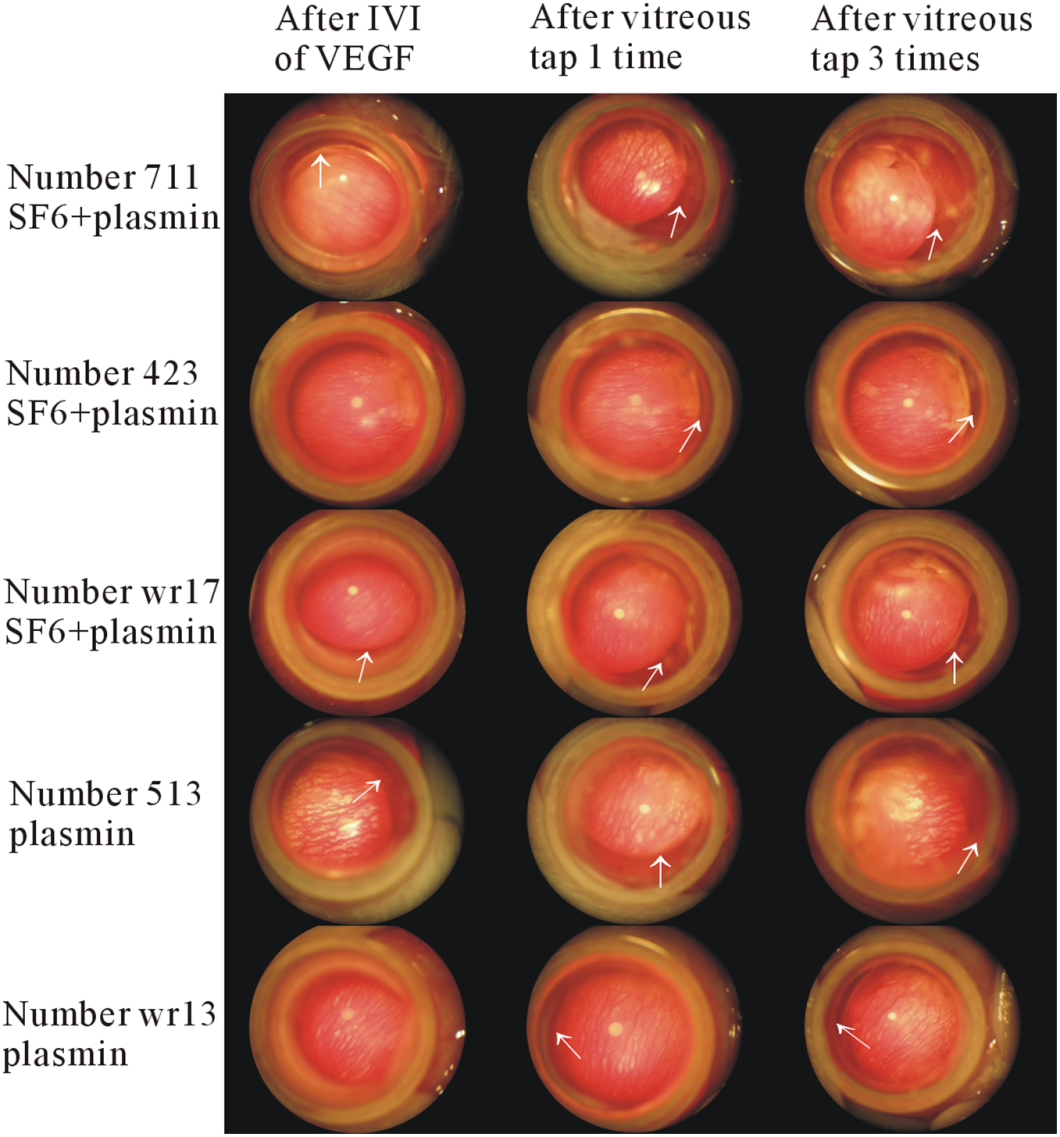
Lens subluxation following plasmin treatment. Representative figures of lens subluxation after intravitreal injections of plasmin and SF_6_, or plasmin alone, with a subsequent high intraocular level of vascular endothelial growth factor (VEGF), and vitreous tap. After intravitreal injections of plasmin with/without SF_6_, VEGF, and vitreous tap, lens subluxation was noted in the eyes treated with plasmin and SF_6_ (plasmin+SF_6_ group) or plasmin alone (plasmin group) (white arrows). Lens subluxation was progressive in some cases (case number 711, wr17, and wr13) and stationary in others (case 423, and 513). IVI, intravitreal injection.

After treatment, the animals were checked every week to examine whether there was signs of lens subluxation until they were sacrificed (**Figure 2**). The status of lens subluxation in the eyes was verified by two independent observers.

**Figure 3:**
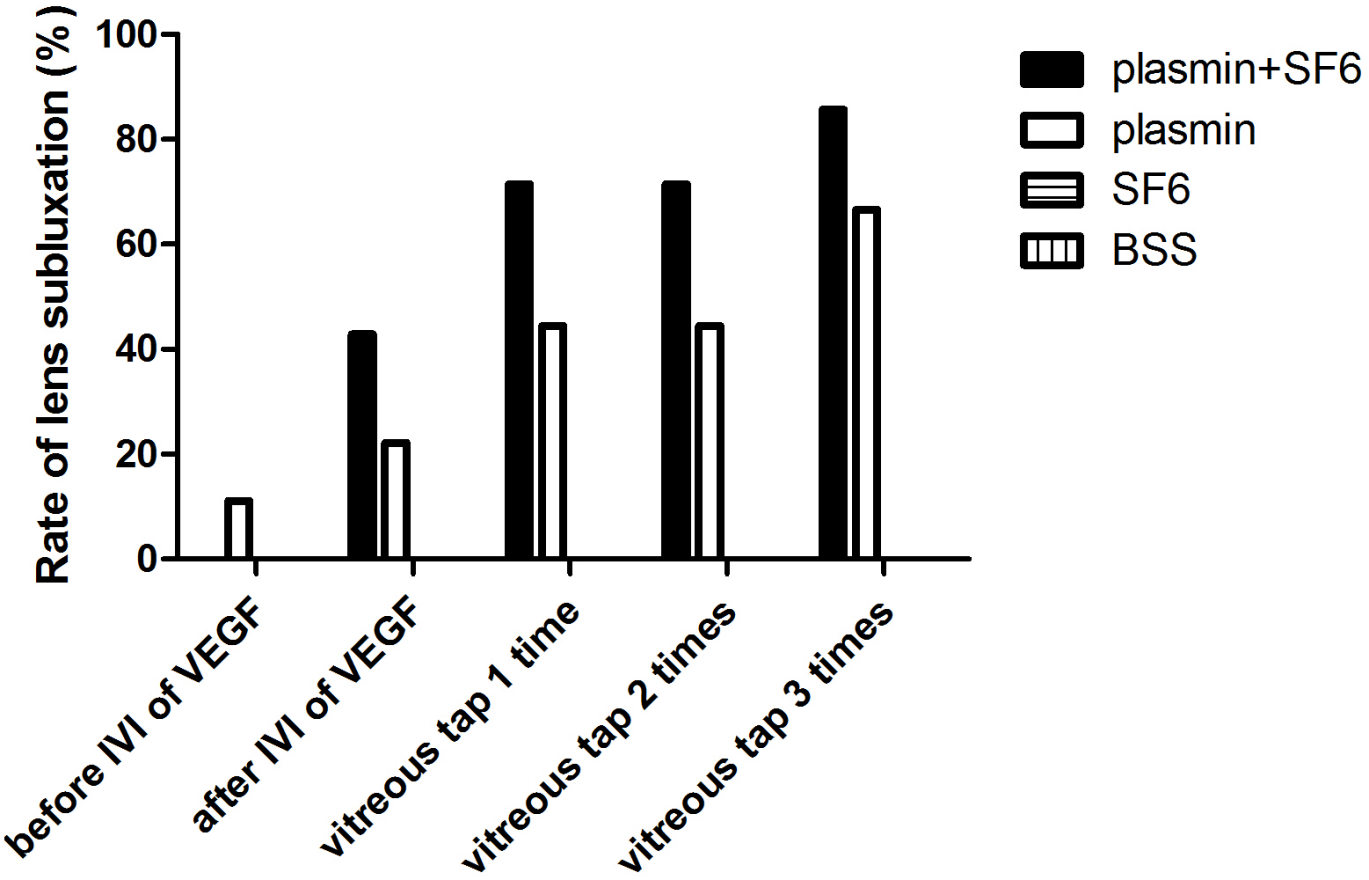
Rate of lens subluxation. Rate of lens subluxation after intravitreal injections of plasmin and/or SF_6_ and subsequent elevation of vascular endothelial growth factor (VEGF) levels, and vitreous tap was performed 3 times is shown. Lens subluxation was noted in the eyes treated with plasmin before the injection of VEGF. After intravitreal injections of VEGF, and vitreous tap was performed 3 times, lens subluxation increased in the eyes treated with plasmin and SF_6_ (plasmin+SF_6_ group) or plasmin alone (plasmin group). The eyes treated with SF_6_ (SF_6_ group) or BSS (BSS group) showed no signs of lens subluxation.

Lens subluxation was not found in any rabbit (0%; 0/7 eyes) in the plasmin+SF_6_ group, and one rabbit (11%; 1/9 eyes) in the plasmin group before the intravitreal injection of VEGF. After the intravitreal injection of VEGF, lens subluxation was noted in 43% (3/7) of the eyes in the plasmin+SF_6_ group and 22% (2/9) of the eyes in the plasmin group. Lens subluxation was not found in the other groups (0/7 and 0/8 for the SF_6_ and BSS groups, respectively). After the first vitreous tapping, lens subluxation was noted in 71% (5/7) of the eyes in the plasmin+SF_6_ group and 44% (4/9) of the eyes in the plasmin group. The eyes treated with SF_6_ (SF_6_ group) or BSS (BSS group) showed no signs of lens subluxation (0/7 and 0/8 for the SF_6_ and BSS groups, respectively). After the second vitreous tapping, lens subluxation was noted in 71% (5/7) of the eyes in the plasmin+SF_6_ group and 44% (4/9) of the eyes in the plasmin group. The eyes treated with SF_6_ (SF_6_ group) or BSS (BSS group) still showed no signs of lens subluxation (0/7 and 0/8 for the SF_6_ and BSS groups, respectively). After the third vitreous tapping, lens subluxation was noted in 86% (6/7) of the eyes in the plasmin+SF_6_ group and 67% (6/9) of the eyes in the plasmin group. The eyes treated with SF_6_ (SF_6_ group) or BSS (BSS group) again showed no signs of lens subluxation (0/7 and 0/8 for the SF_6_ and BSS groups, respectively). The data are shown in **Figure 3**. Based on the results of GEE, the degrees of lens subluxation in the plasmin+SF_6_ and plasmin groups were significantly different from the SF_6_ group (p<0.0001 and p = 0.035, respectively) and BSS group (p<0.0001 and p = 0.033, respectively). In the analysis of the severity of lens subluxation, 11 eyes (92%) had mild subluxation and 1 eyes (8%) in the plasmin+SF_6_ group had moderate subluxation out of the 12 eyes with lens subluxation. None of the eyes developed severe subluxation following intravitreal injections.

**Figure 4:**
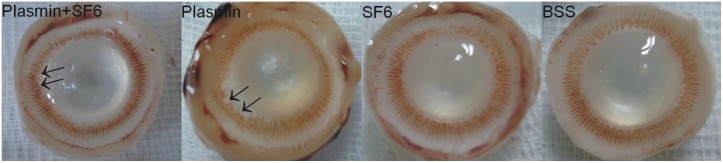
Ciliary process disruption after plasmin treatment. Ciliary process disruption after intravitreal injections of plasmin. Viewed from the back of the eye, partial ciliary body disruption was noted in the eyes treated with plasmin and SF_6_ (plasmin+SF_6_ group) or plasmin alone (plasmin group) (black arrows). The eyes treated with SF_6_ (SF_6_ group) or BSS (BSS group) showed no signs of ciliary process disruption.

On tissue sectioning, partial ciliary body disruption was noted in all of the eyes treated with plasmin with or without SF_6_ (plasmin+SF_6_ and plasmin groups). The eyes treated with SF_6_ (SF_6_ group) or BSS (BSS group) showed no signs of ciliary process disruption (**Figure 4**).

**Figure 5:**
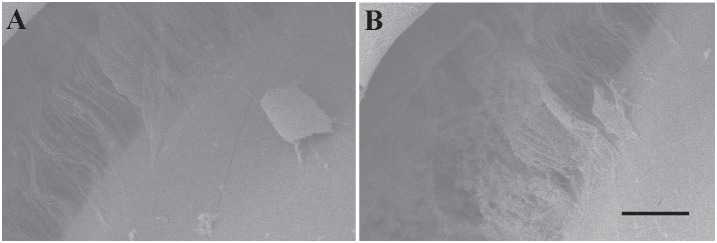
Zonular fiber disruption after plasmin treatment. Zonular fiber disruption after intravitreal injections of plasmin in scanning electron microscopy (SEM). The SEM findings showed that the zonular fibers remained intact in the control eyes (A), however zonular fiber disruption was noted in the eyes treated with plasmin (B). Scale: 400 μm.

On the scanning electron microscope examinations, zonular disruption was confirmed in the eyes treated with plasmin and with lens subluxation, while the zonular fibers remained intact in the eyes without plasmin treatment (**Figure 5**).

## Discussion

Lens subluxation, which may be congenital, developmental, or acquired, is usually caused by abnormalities of or injury to a portion of the suspensory ligaments, the zonular fibers which anchor the lens to the ciliary muscle. Etiologies of lens subluxation include blunt ocular trauma, pseudo-exfoliation, high myopia and congenial diseases such as Marfan syndrome, homocystinuria and others [36]. Visual disturbances associated with lens dislocation include monocular double vision, decreased vision, and astigmatism. Our findings showed that the incidence of lens subluxation increased following plasmin injection, with a high VEGF level, and vitreous tap. Zonular fibers and the ciliary body may therefore be affected and disrupted following those events. For the eyes without plasmin treatment, no lens subluxation was encountered even with a high VEGF level and vitreous tap. These findings suggest that plasmin could act on and weaken the zonular fibers, thereby causing lens subluxation. Previously, plasmin enzyme was extracted autologously [11]–[17]. However, a commercially available truncated plasmin, ocriplasmin, has recently been approved for use in vitreomacular traction and macular holes, and careful selection of patients has been shown to lead to resolution of vitreomacular traction and macular hole closure in 50% of patients [26], [37]. As previously stated, two pre-marketing cases of lens subluxation following ocriplasmin treatment have been reported. Since ocriplasmin and plasmin act via the same active site, this effect may be of particular concern when using plasmin or plasmin-associated enzymes in patients with vitreomacular abnormalities. Long-term follow up of these patients is needed to monitor this possible complication.

In this study, there was no subluxation in the SF_6_ group, whereas 43%–86% lens subluxation was noted in the SF_6_+plasmin group following various treatments. A possible explanation for this finding is that both SF_6_ and plasmin treatment could cause zonule damage, however plasmin had a far more damaging effect on the zonular fibers than SF_6_. Therefore, SF_6_ treatment alone did not cause clinically visible lens subluxation, but its use in combination with plasmin enzyme, which can digest the zonules directly and have a higher impact on zonular integrity, led to a higher proportion of cases developing lens subluxation, a rate higher than plasmin treatment alone.

Tractional force from the vitreous has been found to be a major factor associated with a variety of vitreoretinopathies, including macular hole, epiretinal membrane, diabetic retinopathy, and retinopathy of prematurity. Plasmin enzyme has been found to be an effective agent to cleave the vitreoretinal juncture and weaken vitreoretinal adhesion and help to produce PVD. Therefore the use of plasmin may help to treat these diseases [11]–[17]. Combining SF_6_ with plasmin has been reported to cause PVD even more effectively, which is useful for situations with a tougher vitreoretinal juncture [38].

Intrinsic components of zonular fibers have been identified by biochemical and immunohistochemical techniques. Fibrillar components including at least eight different microfibrillar glycoproteins [39], and fibrillin, a component of microfibrils and microfibril-associated glycoprotein, have been found on the beaded structures of zonular fibers [40]. Fibrillin was later identified to be a 350 kDa glycoprotein which is coded for by a gene on chromosome 15 q21.1 [41], [42]. Classic Marfan syndrome with ectopia lentis has been linked to mutations of the fibrillin gene [41]. The zonular fibers are surrounded by a coating consisting of non-fibrillar components including glycosaminoglycans (hyaluronan) [39], proteoglycans [39], laminin [24], and fibronectin [25]. In addition, the ciliary body has been reported to express laminin and fibronectin [10]. Therefore, plasmin may act on ciliary zonules and the ciliary body because laminin and fibronectin are expressed in these structures.

VEGF has been found to be elevated in eyes with a variety of retinopathies [27]–[34]. Anti-VEGF has therefore become a major treatment option for these retinopathies with promising results. VEGF has also been shown to be the principle factor for angiogenesis [43], and proteolysis has been reported to be one of the first and most sustained activities involved in VEGF-mediated angiogenesis [44]–[46]. It is likely that an elevated level of VEGF can cause inflammation in the eyes, and that this could trigger a proteolytic effect on zonular fibers and even ciliary processes, thus reducing the strength of the zonular fibers and facilitating lens subluxation.

In eyes that have undergone intraocular surgery or ocular trauma, such as vitreous tap, the zonular fibers may also be affected by mechanical force. The zonular fibers are delicate fibers with a size of only 10 nm [47], and the tractional effect caused by vitreous tapping could potentially affect the integrity of the zonular fibers, thus causing lens subluxation.

Previous studies have shown a maximal plasmin enzymatic activity between 15 and 60 minutes after injection [21], [22], decreasing thereafter and being either undetected after 24 hours [22] or detected at a very low level [21]. Because of the relatively short half-life of plasmin in the vitreous, the use of this enzyme seems to be well tolerated in the eye because it degrades in a short time following injection. Although plasmin has been safely used in clinical settings, some potential complications can arise with its use. Transient decreases in electroretinogram [22] and mild intraocular inflammation [22], [48] following intravitreal injections of plasmin have been reported. Posterior subcapsular cataracts have also been noted in some patients with pediatric traumatic macular holes following plasmin-mediated vitrectomy [17], [49]. According to the results of this study, zonule weakness and lens subluxation or dislocation may be another potential complication associated with the use of this enzyme.

Clinically, there are two ways of applying plasmin enzyme: intravitreal injection only, and the combined use of plasmin and vitrectomy (plasmin-assisted vitrectomy). In plasmin-assisted vitrectomy, the injection of plasmin enzyme usually takes place 30 minutes before the start of vitrectomy. The likelihood of developing lens subluxation may be reduced in this scenario because the enzyme can be washed out during vitrectomy. In the case of intravitreal injection only, the patients may adopt a supine position to settle the enzyme on the posterior retina following the injection to facilitate enzymatic degradation on the vitreoretinal junction and decrease the likelihood of contact with the zonular fibers.

In the current study, the VEGF injection was not performed before the injection of plasmin and/or SF_6._ Instead, plasmin and/or SF_6_ were injected first, followed by the injection of VEGF and vitreous tapping. We used this method because we were curious about the effect of plasmin enzyme on zonular integrity and whether further increases in the high level of VEGF in the eye such as retinal vessel occlusion and vitreous tap would further aggravate the condition. In a clinical setting, plasmin or ocriplasmin can be used in eyes with high levels of VEGF such as in diabetic retinopathy. We recognize that our study design may be somewhat different from such a clinical setting, however we do not think that this detracts from our results. Plasmin may have a different impact on fibronectin and laminin compared with ocriplasmin, although both of them can produce PVD effectively. In addition, while an exogenous VEGF injection was used to mimic increased endogenous VEGF levels, the dose of 500 ng per eye used in the current study is higher than that usually found in age-related macular degeneration or proliferative diabetic retinopathy [50], [51]. Nevertheless, these factors are limitations of this study which should be taken into consideration when interpreting the higher number of cases of lens subluxation seen in the current study.

In conclusion, plasmin enzyme can weaken zonular fibers and disturb ciliary processes, thereby causing lens subluxation. A high level of VEGF in the vitreous and mechanical trauma caused by vitreous tap could facilitate such events. These results imply a potential complication associated with the clinical use of this enzyme for patients with vitreomacular abnormalities.
